# Effect of Gastrointestinal Surgical Manipulation on Metabolic Syndrome: A Focus on Metabolic Surgery

**DOI:** 10.1155/2012/670418

**Published:** 2012-10-22

**Authors:** Mario Rizzello, Francesco De Angelis, Fabio Cesare Campanile, Gianfranco Silecchia

**Affiliations:** ^1^Division of General Surgery, Department of Medico-Surgical Sciences and Biotechnology, Hospital ICOT, Sapienza University of Rome, via F. Faggiana 1668, 04100 Latina, Italy; ^2^Division of General Surgery, Hospital of Civita Castellana, via Ferretti 169, 01033 Civita Castellana (VT), Italy

## Abstract

Metabolic syndrome is strictly associated with morbid obesity and leads to an increased risk of cardiovascular diseases and related mortality. Bariatric surgery is considered an effective option for the management of these patients. We searched MEDLINE, Current Contents, and the Cochrane Library for papers published on bariatric surgery outcomes in English from 1 January 1990 to 20 July 2012. We reported the effect of gastrointestinal manipulation on metabolic syndrome after bariatric surgery. Bariatric surgery determines an important resolution rate of major obesity-related comorbidities. Roux-en-Y gastric bypass and biliopancreatic diversion appear to be more effective than adjustable gastric banding in terms of weight loss and comorbidities resolution. However, the results obtained in terms of weight loss and resolution of comorbidities after a “new bariatric procedure” (sleeve gastrectomy) encouraged and stimulated the diffusion of this operation.

## 1. Introduction

In 2008 there were worldwide an estimated 1.5 billion adults overweight and 500 million obese. More than 40 million children are estimated to be overweight. Obesity rates have more than doubled since 1980, with 1 in 10 of the world's adult western population now obese. Morbid obesity (BMI > 35) is responsible for more than 2.5 million deaths per year worldwide [1], and it has been estimated that life expectancy of a 25-year-old morbidly obese man is 12 years lower because of this condition. 

In morbidly obese patients, metabolic syndrome (MetS) is a constellation of metabolic abnormalities, including type 2 diabetes mellitus (T2DM), hypertension, dyslipidemia, and ovarian polycystic syndrome, that lead to an increased risk of cardiovascular diseases and related mortality [2]. The physiopathology of MetS is not completely known, but there is an intertwined link between obesity (BMI > 30), insulin resistance, and the MetS, which leads to a vicious cycle of metabolic stress with relevant clinical pattern [3]. Moreover, it has long been assumed that the presence of MetS is a risk factor for adverse outcomes in patients undergoing bariatric/metabolic surgery. In fact, in obese patients with MetS, central and visceral adiposity and hepatomegaly make bariatric surgery more technically challenging. Furthermore, in these patients MetS leads to a heightened state of systematic inflammation, with consequent lower ability to face the stress of bariatric surgery and limitations of body's response to complications [4].

We describe the effects of the main laparoscopic bariatric/metabolic procedures on MetS and the mechanisms underlying these effects.

## 2. Laparoscopic Bariatric Procedures

### 2.1. Adjustable Silicone Gastric Banding

Laparoscopic adjustable silicone gastric banding (LAGB) was the first bariatric procedure to be performed by a laparoscopic approach. Introduction of LAGB into clinical practice was an immediate success in Europe as well as in Australia. Although standard Roux-en-Y Gastric by Pass (RYGBP) and Biliopancreatic Diversion with Duodenal Switch (BPD-DS) currently represent the majority of laparoscopic bariatric/metabolic procedures in the United States and Canada, in USA laparoscopic gastric restrictive procedures during the last 5 years have been growing acceptance by physicians as well as by patients.

**Figure 1:**
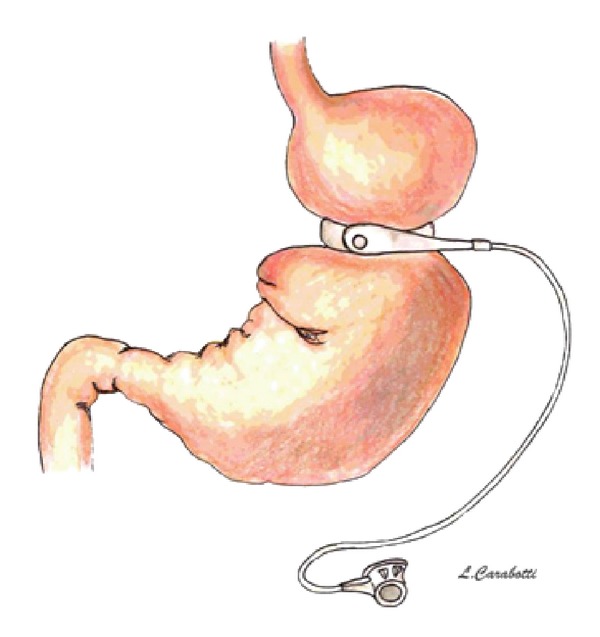
Adjustable gastric banding.

The idea behind the operation is to “create” a small pouch in the upper part of the stomach with a controlled and adjustable stoma, without stapling, thus limiting the daily food intake (restrictive procedure). The silicone prosthesis is fitted around the stomach just below the gastroesophageal junction, creating a 15–20 mL pouch (virtual pouch) (Figure 1). This operation does not involve neither rerouting of food through the upper gastrointestinal tract nor exclusion of intestinal segments. The weight loss process in the short and long term is due to the food intake restriction and early satiety. 

The MetS modifications after LAGB occur gradually and are related with the degree of weight loss. In the highest-quality study, excess body weight loss at 1 year after LAGB is 48%. At this time the hypertension, diabetes, dyslipidemia, and sleep apnea resolution rate were about 55%, 58%, 42%, and 45%, respectively [5]. Weber et al. reported an EWL of 42.1% at 24 months with a hypertension, diabetes, and dyslipidemia resolution rate of 70%, 60% and 0%, respectively. At a followup of 3 years, Cottam et al. reported an average BMI-loss of 16 Kg/m^2^ with hypertension, diabetes, hypercholesterolemia, and hypertriglyceridemia resolution rate of 56%, 50%, 40%, and 46%, respectively. In adolescent obese patients, at 5 years followup after LAGB, Silberhumer et al. reported an EWL of 92.6% with a complete resolution of hypertension, diabetes, and dyslipidemia [6].

In a randomized controlled trial, Dixon et al. analyzed 60 obese patients (BMI > 30 and < 40) with recently diagnosed (<2 years) T2DM. The patients were randomized in conventional diabetes therapy with a focus on weight loss by lifestyle change versus laparoscopic adjustable gastric banding with conventional diabetes care. The primary end point was remission of T2DM and secondary end points included weight loss and components of the MetS. Diabetes remission was achieved by 22 patients (73%) in the surgical group and 4 patients (13%) in the conventional-therapy group. Remission of T2DM was related to weight loss and lower baseline HbA1c levels. This study demonstrates superior glycemic control and diabetes remission rates with adjustable gastric banding through greater weight loss. After 2 years, the surgical group displayed a 5 times higher remission rate and 4 times greater reduction in HbA1c values than the conventional-therapy group [7].

### 2.2. Roux-en-Y Gastric Bypass

**Figure 2:**
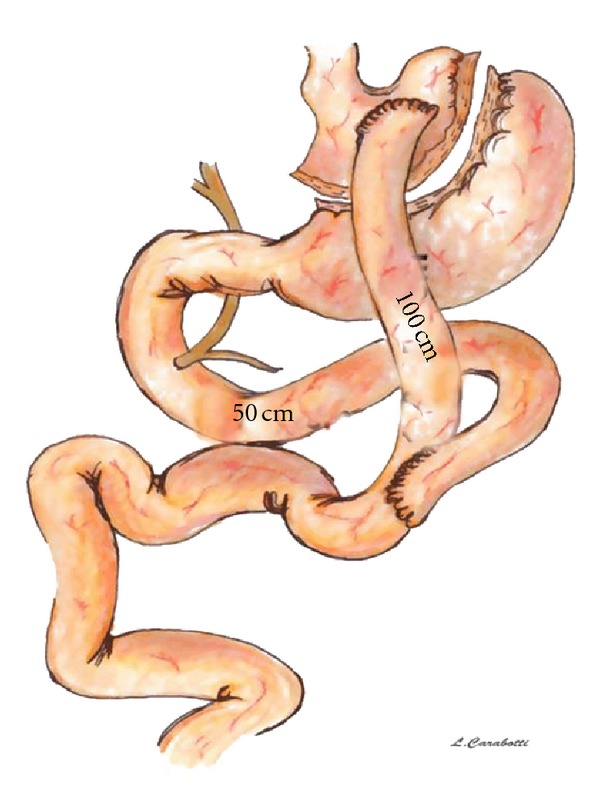
Roux-en-Y gastric bypass.

Laparoscopic Roux-en-Y gastric bypass with isolated gastric pouch was described in 1993 by Wittgrove et al. The RYGB is the most largely performed bariatric/metabolic procedure in the USA. It is estimated that 220,000 such operations were performed in the United States in 2010 (Figure 2). 

The standard gastric bypass procedure consists in

- creation of a small, (15–30 mL) isolated gastric pouch using an endoscopic surgical stapler, accompanied by a bypass of the remaining stomach, duodenum, and first tract of jejunum;
- reconstruction of the GI tract with the Roux limb with a biliary loop length of 30–75 cm and alimentary limb length of 100–150 cm.

In the variant “long limb,” the length of the alimentary limb is 150–250 cm; in the “distal” RYGB, the common channel length is 150 cm, measured from ileocecal valve. The latter variant is more similar to the BPD inducing more intestinal malabsorption than standard LRYGB, which produces a limited malabsorption of around 30% of lipid. 

In a high-quality study excess body weight loss at 1 year was 76% after RYGB. Blood pressure decreases significantly after this procedure and it has been shown that at 1 year of followup 46% of patients achieved complete resolution of hypertension while 19% showed an improvement. In addition, the patients with complete resolution had a shorter diagnosis interval of hypertension compared with patients without resolution (53 versus 95 months, resp., *P* = 0.01). 

The RYGB prevents diabetes in 99-100% of patients with impaired glucose tolerance and leads to clinical resolution of 80–90% of newly diagnosed cases of T2DM. 

Moreover, after RYGBP, a rapid improvement in insulin resistance within few days has been described. Wickremesekera reported changes in insulin resistance following gastric bypass (GBP) and demonstrated a rapid improvement in insulin resistance within 6 days of surgery (Δ homeostatic model assessment for insulin resistance (HOMA IR) 7.3) [8]. Ballantyne et al. reported, at 3 months, a postoperative HOMA IR level significantly lower following GBP than after Laparoscopic Adjustable Gastric Banding (LAGB) [9].

Higa et al. found that 83% of diabetic patients had a resolution or improvement after RYGB, and this was maintained in 67% of patients at 10 years of followup. Pories reported a diabetic resolution rate of over 80% after a maximum followup of 14 years. Sugerman reported good results in the long-term, with 83 and 86% having resolution of diabetes at 1 and 5–7 years, respectively [10]. The SOS study, however, reported an increase in-incidence of diabetes at 10-year followup compared with 2-year followup [11].

Schauer et al. reported the results of a retrospective study in order to define the effect of laparoscopic gastric bypass on type 2 diabetes and the predictors of diabetes resolution. On 191 patients with IGT or type 2 diabetes that underwent a gastric bypass, fasting plasma glucose and glycosylated haemoglobin concentrations returned to normal levels (83%) or markedly improved (17%) in all patients. A significant reduction in the use of oral antidiabetic agents (80%) and insulin (79%) followed surgical treatment. Patients with the shortest duration (<5 years), the mildest form of T2DM (diet controlled), and those with the greatest weight loss after surgery were most likely to achieve complete resolution of diabetes [12]. Hall et al. showed that patients with a baseline HbA1c > 10% had a 50% rate of remission compared to 77.3% with an HbA1c of 6.5–7.9%. The mean duration of T2DM preoperatively was 5.5 ± 7 years. A preoperative duration of T2DM > 10 years was shown to reduce significantly the chances of remission. The authors stated that a shorter period of time with diabetes and better glycemic control before surgery may result in a successful remission for T2DM, suggesting that bariatric surgery should be performed earlier in diabetic patients [13].

Dyslipidemia and sleep apnea resolution rates are around 65% and 75% at 1 year after RYGBP and are strongly related to the weight-loss and its maintenance in the long term. In a study on RYGBP, a 66% excess weight loss at 12 months postoperatively was associated with a 31% decrease in low-density lipoprotein cholesterol levels, a 39% increase in HDL-C levels, and a 63% decrease in triglycerides [14]. 

Obeid et al. recently reported their results at long-term followup. After 8 years, RYGBP was associated with an EWL of 69%, hypertension, diabetes, dyslipidemia resolution rate of 66%, 82%, and 40%, respectively [15].

### 2.3. Biliopancreatic Diversion

Scopinaro first performed the biliopancreatic diversion (BPD) in 1976 in Genova (Italy). This operation induces controlled malabsorption without many of the potential side effects caused by bacterial overgrowth and indiscriminate malabsorption associated with the Jejuno-Ileal Bypass, which is now completely abandoned.

This operation combines removal of 2/3rd of the stomach (distal gastrectomy) with a long intestinal bypass (common channel 50 cm, alimentary limb 250 cm). The operation includes cholecystectomy and liver biopsy.

The procedure was later modified by Hess with a variant that he called “Duodenal Switch” in 1986 that was first performed laparoscopically by Gagner in 1999.

**Figure 3:**
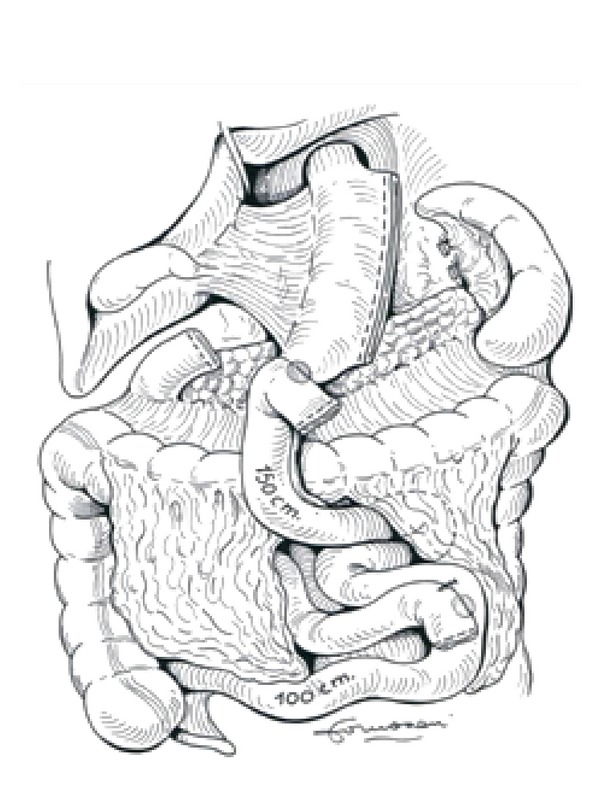
Biliopancreatic diversion with duodenal switch.

Instead of performing a distal gastrectomy, a “sleeve gastrectomy” along the vertical axis of the stomach (volume of remnant 70–150 mL) was proposed, preserving the pylorus and initial segment of the duodenum, which is then anastomosed to a segment of the ileum, similarly to the BPD, to create the alimentary segment (common channel 100 cm). Preservation of the pyloric sphincter is designed to be more physiological. The sleeve gastrectomy decreases the volume of the stomach and also decreases the parietal cell mass, with the intent of decreasing the incidence of ulcers at the duodeno-ileal anastomosis (Figure 3). 

These procedures produce selective malabsorption by limiting food digestion and absorption to a short, common ileal segment. The potential for nutritional complications exists. Patients undergoing the biliopancreatic diversion or duodenal switch procedure require close long-term medical followup and regular monitoring of fat-soluble vitamins, vitamin B12, iron, and calcium.

Scopinaro et al. report the long-term outcome of BPD in a series of 312 obese patients with T2DM. Fasting serum glucose concentration fell to within normal values in all but two of the patients and remained in the physiological range in all but six, for a mean followup of 10 years [16].

Scopinaro's group has also reported reduced HOMA values, and, by inference, improved insulin sensitivity, four days after BPD (Δ HOMA IR 4.6) [17]. The improvement after GBP and BPD was clearly unrelated to weight loss, which proceeded much slower. These data suggested that GBP and BPD improve diabetes through a hormonal effect on the enteroinsular axis [18].

Inabnet reported recently a hypertension and dyslipidemia resolution rate of 52.9% and 64%, respectively, after BPD-DS.

### 2.4. Sleeve Gastrectomy

**Figure 4:**
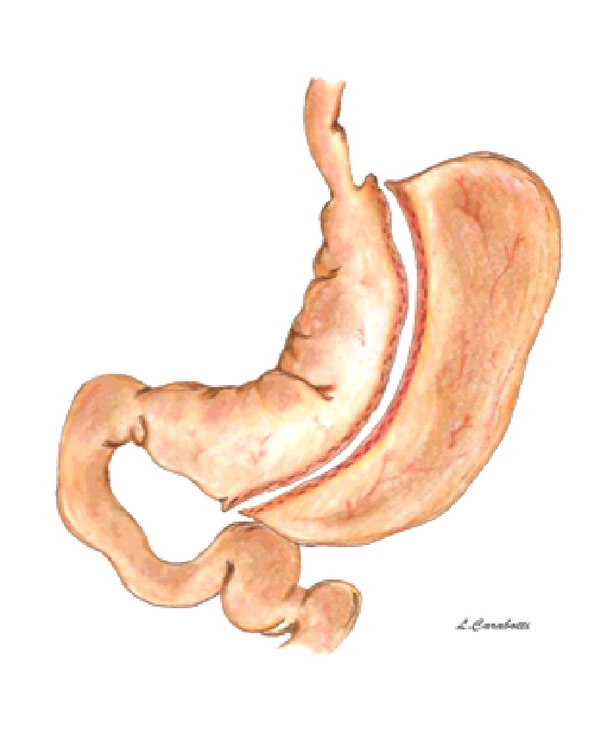
Sleeve gastrectomy.

In order to reduce operative morbidity and mortality in high-risk superobese patients, BPD-DS was divided in two stages: laparoscopic sleeve gastrectomy (LSG) as first stage followed after 6–12 months and by second stage consisting in duodeno-ileostomy and ileo-ileostomy (Figure 4).

Results obtained in terms of weight loss and resolution of comorbidities after LSG encouraged and stimulated the diffusion of this operation inducing several Authors to propose this procedure as a primary bariatric procedure. In fact, LSG is a technically simple surgical procedure with a low complication rate and negligible long-term nutritional deficiencies.

The effect on weight loss and resolution of comorbidities has been attributed to the reduction of the gastric capacity (restrictive effect) and/or to the orexigenic and anorexigenic intestinal hormones modification (hormonal effect).

Silecchia et al. demonstrated that LSG reduce the operative risk in superobese patients undergoing two-stage BPD-DS achieving marked weight loss as well as significant reduction of major obesity-related comorbidities. 41 super-obese high-risk patients (mean BMI 57.3 ± 6.5 kg/m^2^, age 44.6 ± 9.7 years) were entered into a prospective study and 9 had BMI > 60. Type 2 diabetes/IGT was registered in 17 patients (41%). At surgery, 41.5% were classified ASA 4 and 58.5% as ASA 3 (mean ASA score 3.4 ± 0.5). After 12 months, mean ASA score was 2.7 ± 0.8 (*P* < 0.001). At 18th month following SG, diabetes/IGT was cured in 76.9% and improved in 15.4% of patients. Hypertension was cured in 62.5% and improved in 25% of patients; OSAS was cured in 56.2% end improved in 32.2% of patients [19]. Rizzello et al. reported a marked and very early (3–5 days) reduction in HOMA IR in diabetic patients, thus indicating a rapid and remarkable improvement of insulin sensitivity after SG (Δ HOMA IR 13.9 ± 1.2) unrelated to weight loss [20]. Abbatini et al. in a retrospective study including 110 morbid obese patients with type 2 diabetes/Impaired glucose tolerance who had bariatric surgery (45 LAGB, 45 LRYGB, 20 LSG) showed that surgery controlled T2DM/IGT in 74.5% of patients at 3-year followup. Antidiabetic drugs were discontinued at 12.6 months after LAGB, 3.2 and 3.3 months after LRYGB and LSG, respectively. Moreover, the efficacy of bariatric surgery in controlling diabetes was strongly related to disease duration [21]. 

### 2.5. New Procedures

**Figure 5:**
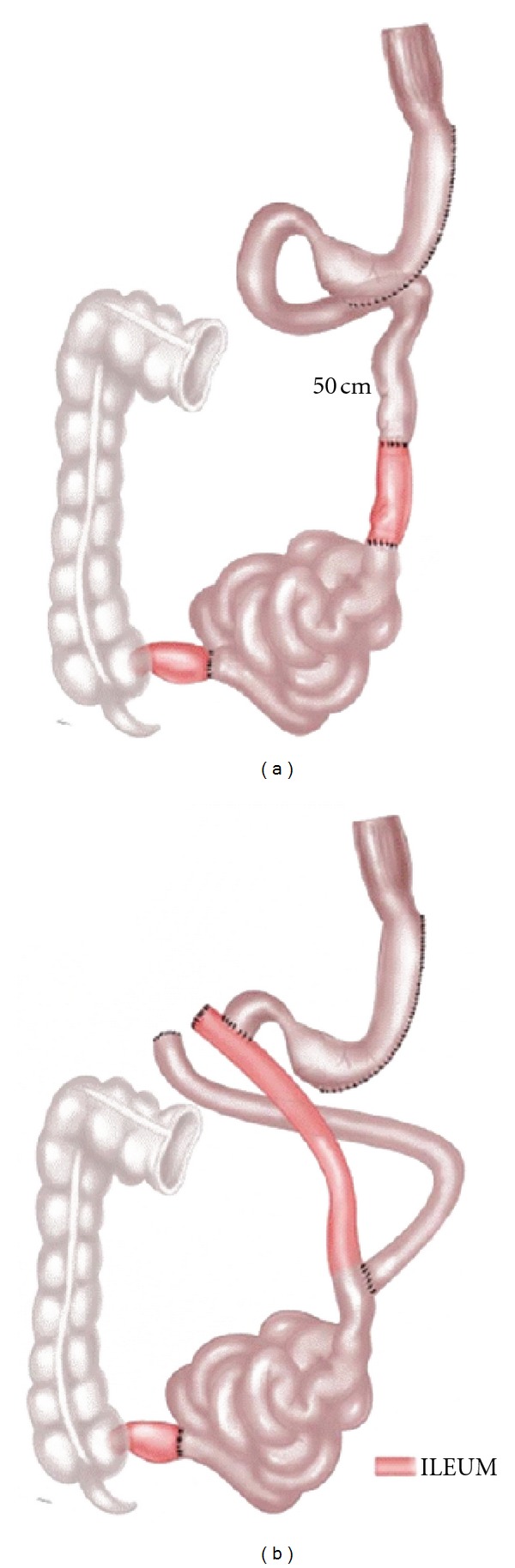
(a) Ileal interposition associated to sleeve gastrectomy. (b) Ileal interposition associated to diverted sleeve gastrectomy.

The “ileal interposition” consists in the transposition and interposition of an isolated segment of ileum to the jejunum. The first technique described by DePaula et al. started with division of the jejunum 30 cm from the ligament of Treitz using a linear stapler. An ileal segment of 150 cm was created 50 cm proximal to the ileocecal valve, interposed peristaltically into the proximal jejunum. Ileal interposition was associated to a sleeve gastrectomy. The second technique was an ileal interposition associated with a diverted LSG. LSG was performed and the duodenum was transected using a 60 mm linear stapler. An ileal segment of 150 cm was created 50 cm proximal to the ileocecal valve, interposed and anastomosed peristaltically to the proximal duodenum. A point in the jejunum 50 cm from the ligament of Treitz was measured and anastomosed to the distal part of the interposed ileum. These procedures were performed by laparoscopy (Figure 5) [22].

**Figure 6:**
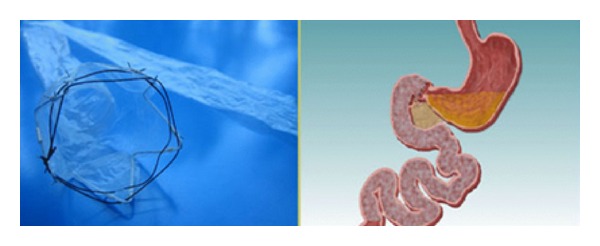
The EndoBarrier gastrointestinal liner. Food bypasses the duodenum and proximal jejunum as it does in a Roux-en-Y Gastric Bypass.

The potential use of endoluminal techniques in the field of bariatrics has prompted investigation into several promising applications. The technology currently under development can be divided roughly into four categories: suturing and stapling devices, endoluminally delivered prostheses, ablation-based devices, and electrical stimulation-based devices. In particular, the placement in duodenum of a prosthetic tube to prevent the contact of nutrients with the duodenal-jejunal mucosa may reproduce the same effect of RYGB/BPD in diabetes resolution (Figure 6).

## 3. Bariatric Surgery and Diabetes Control

Morbid obesity is associated with insulin resistance and marked insulin hypersecretion, but the function of *β*-cell is preserved. On the other hand, diabetes and impaired glucose tolerance are characterized by a progressive loss of *β*-cell glucose sensitivity, independent of insulin resistance. Bariatric surgery leads to an improvement in insulin sensitivity and decrease of insulin secretion [23]. The duration and severity of T2DM also appear to be key factors for its remission after bariatric surgery. Hall et al. showed that patients with a baseline HbA1c > 10% had a 50% rate of remission, after RYGBP, compared to 77.3% with an HbA1c of 6.5–7.9%. The mean duration of T2DM preoperatively was 5.5 ± 7 years. A preoperative duration of T2DM > 10 years was shown to significantly reduce the chances of remission. The authors concluded that a shorter period of time with diabetes and better glycemic control before surgery may result in a better remission rate for T2DM, suggesting that bariatric surgery should be performed earlier in diabetic patients. Obese T2DM patients undergoing RYGB were more likely to achieve full remission if duration of disease was inferior to 5 years and/or glycemia was controlled only through diet). The remission rate in patients with T2DM for ≤5 years was 95% compared to 75% in patients who had diabetes for 6 to 10 years and 54% in those who had diabetes for more than 10 years (*P* < 0.001) [13].

 A possible explanation for this observation is that the compensatory and inflammatory process of insulin resistance leads to the deterioration and loss of the pancreatic *β* cells through increased apoptosis. However, there is a reversible component of *β* cells deterioration, with weight loss improving *β* cells responsiveness to glucose [24, 25]. The practice of early bariatric surgery before an irreversible *β* cell failure could increase the probability of T2DM remission.

There have been many hypotheses concerning the mechanism of surgical resolution of diabetes. Weight loss, decreased caloric intake, malabsorption, the early delivery of nutrients to the distal small intestine, and the exclusion of the proximal small bowel have all been proposed as possible explanations. Moreover, euglycemia and normal insulin levels occur within days after surgery, long before any significant weight loss [26].

In 2004, Rubino and Marescaux reported an experimental study that support the hypothesis that the bypass of duodenum and jejunum can control type 2 diabetes and directly and not secondarily to weight loss or treatment of obesity. A gastrojejunal bypass (GJB) with preservation of an intact gastric volume was performed in 10- to 12-week-old Goto-Kakizaki rats, a spontaneous nonobese model of type 2 diabetes. GJB significantly improved glycemic control. After surgery blood glucose was controlled better than after greater weight loss from food restriction or rosiglitazone therapy. These effects were not seen in the sham-operated animals despite similar operative time, same postoperative food intake rates, and no significant difference in weight gain profile [27].

Two hypotheses have been proposed to explain the RYGB effect on the glucose homeostasis. The “hindgut hypothesis” holds that diabetes control results from the expedited delivery of nutrient chime to the distal intestine, enhancing a physiologic signal that improves glucose metabolism. The incretin glucagon-like peptide-1 (GLP-1) is implicated in this hypothesis as the L cells located primarily in the distal ileum and colon secrete it. GLP-1 induces insulin secretion in response to glucose and satiety, likely through action on hypothalamic and vagal receptors. Early stimulation of these cells after RYGB/BPD/DS could result in increased GLP-1 production and consequent enhanced insulin secretion. The role of GLP-1 on diabetes remission seems to be confirmed by the “ileal interposition” that causes an accelerate delivery of nutrients to the GLP-1 producing areas of intestine. Ileal interposition effectively improves glucose tolerance and it is associated with dramatically elevated ileal hormones, GLP-1, PYY, and glucagon in rats. Early results of ileal interposition on diabetic human patients seem to confirm the experimental data [22, 28].

The alternative hypothesis (“foregut hypothesis”) is that the exclusion of the duodenum and proximal jejunum from the transit of nutrients may prevent secretion of a putative signal that promotes insulin resistance and type 2 diabetes. In a rat model, in 2006, Rubino et al. demonstrated that whereas diabetes was controlled excluding the passage of nutrient through the proximal intestine (DJB) the disease would return when that passage was surgically restored. These findings suggest that a proximal intestinal bypass could be considered for diabetes treatment and that potentially undiscovered factors from the proximal bowel might contribute to the pathophysiology of type 2 diabetes. Both human and animal studies show that the prevention of duodenal passage of nutrients improves glucose tolerance only in diabetic patients. This indicates a mechanism likely attributable to aberrant gastrointestinal signalling unique to the diabetic state. This signalling is possibly removed when the proximal intestine is bypassed [29].

**Figure 7:**
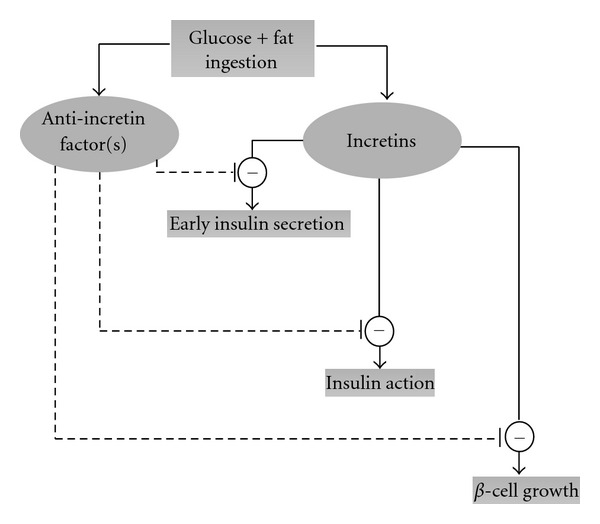
Anti-incretin theory.

The anti-incretin theory proposes that the proximal small bowel produces a hormone or a group of factors, to balance the action of incretin hormones. A dysfunction in the incretin/anti-incretin system, for example the overproduction of anti-incretins, would result in decreased insulin secretion, decreased insulin action (insulin resistance) and a depletion in *β*-cell mass, leading to type 2 diabetes. Correction of this dysfunction in the anti-incretin system by duodenal exclusion may explain the resolution of type 2 diabetes after bypass surgery (Figure 7) [29].

Several studies have also demonstrated a decrease in plasma levels of leptin and insulin, and increased levels of adiponectin and peptide YY3-36 after RYGB and BPD, confirming an endocrine effect of these operations. Analysing 90 morbidly obese patients subjected to laparoscopic bariatric surgery, it was observed the resolution of 91.7% of diabetes, 87.3% of impaired glucose tolerance and 100% of impaired fasting glucose. The authors demonstrated an increase of adiponectin plasma levels and the improvement of insulin-sensitivity measured by the euglycemic hyperinsulinemic clamp technique (M index) [30]. 

Sleeve gastrectomy (SG) has recently emerged as a new “food limiting” bariatric procedure. Data from case series have shown that SG is associated with a high rate of resolution of type 2 diabetes and other obesity-associated comorbidities such as hypertension, hyperlipidemia, and sleep apnea. Vidal et al. showed that at 12 months after surgery, SG is as effective as RYGB (84.6% in both groups) in inducing remission of type 2 diabetes and the metabolic syndrome [31]. In a prospective double-blind study, Karamanakos compares the effects of RYGB with SG. The results showed that PYY levels increased similarly after either procedure. 

**Figure 8:**
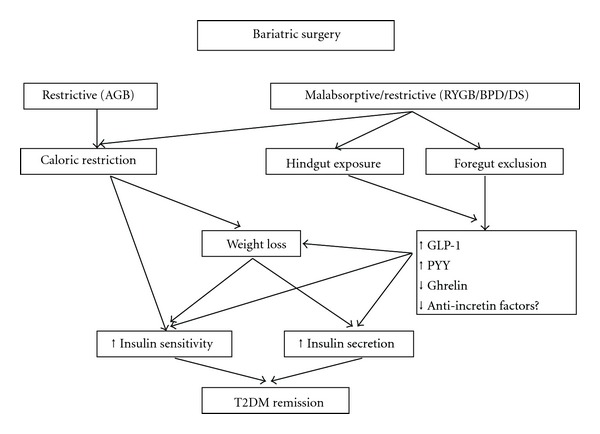
Proposed model for mechanisms of T2DM resolution after bariatric surgery (simplified from [32]).

These data seem to suggest that SG is not a simple restrictive procedure but a hormonal mechanism involved in weight loss and diabetes remission. The markedly reduced ghrelin levels in addition to increased PYY levels after LSG are associated with greater appetite suppression and excess weight loss compared with LRYGB [33] (Figure 8). Peterli et al. analyzed the fasting and test meal-stimulated GLP-1, PYY, and ghrelin modifications after GBP and SG. A statistically significant reduction in fasting ghrelin concentrations was observed in both procedures. After a standard test meal, an early (1 week) significant increase in the GLP-1 and PYY area under the curve (AUC) and a significant decrease in the ghrelin AUC were reported in both procedures. The authors rejected the idea that the proximal small intestine mediates the improvement in glucose homeostasis after bariatric surgery [34]. Recently, Pacheco et al. reported a restoration of the first phase of insulin secretion and improved insulin sensitivity in diabetic obese patients immediately after SG, before any food passage through the gastrointestinal tract, before any weight loss, related to ghrelin, GLP-1, and PYY hormonal changes neither meal- nor weight-change-related [35]. Duration of the disease up to 10 years seems to be a major cut-off in the pathophysiological changes induced by SG.

## 4. Surgery for Diabetes in Nonmorbid Obese Patients

Similar to the procedure described in Rubino, a study by Pacheco et al. in nonobese diabetic rats Goto-Kakizaki (GK) found that duodenal-jejunal exclusion decreased fasting glucose and glucose levels during an OGTT by 1 week after surgery compared to not operated control GK rats. There was no effect of the procedure on glucose-stimulated GLP-1 or GIP levels compared to controls; however, there was a significant decrease in glucagon and leptin levels following the glucose load 1 week after surgery [35].

Another study in GK rats showed that glucose tolerance improved during an OGTT 30 days after ileal interposition. Plasma GLP-1 levels during the first 15 min of the OGTT, measured 45 days after ileal interposition, were significantly increased compared to sham-operated controls. The same group, later, also reported increased insulin levels during the OGTT with increased insulin sensitivity 5months after ileal interposition compared to control groups [36]. Strader et al. showed that ileal interposition effectively improves glucose tolerance in streptozocin-diabetic rats (Long-Evans) and euglycemic rats. By 11 weeks after surgery, glucose and insulin tolerance were markedly improved in interposed-diabetic compared to sham-diabetic rats [28]. 

What is the BMI cut-off for considering surgery to treat diabetes? The “diabetes surgery” should be considered regardless of BMI ranges. Other parameters should be available for surgical intervention: diabetes disease duration, medical therapy (oral hypoglycemic versus insulin), and pancreatic insulin reserve.

Although there is wide scientific evidence that bariatric surgery is effective in providing partial or complete remission for morbid obese patients with diabetes and surgery for morbid obesity is cost effective especially when the patient has type 2 diabetes, nevertheless the American Society for Metabolic and Bariatric Surgery (ASMBS) believes there is insufficient evidence on surgery for type 2 diabetes in nonobese patients (BMI < 30) and it should be limited to approved clinical research studies.

Several Brazilian studies show the efficacy of gastrointestinal surgery on diabetes control in nonobese patients but with short follow-up results. Recently, DePaula et al. published a study including 69 patients with type 2 diabetes mellitus and BMI between 21 and 29 who had laparoscopic “ileal interposition” combined with a sleeve gastrectomy. Overall, 95.7% of the patients achieved adequate glycemic control (HbA1c < 7%) without antidiabetic medication with a mean postoperative followup of 21.7 months (range 7–42 months) [22].

In another study, twenty diabetic patients with BMI < 30 underwent laparoscopic duodenal-jejunal exclusion, with a significant reduction in fasting glycaemia (43.8%) and HbA1c (22.8%) at six months. At this time, only two patients were on oral medication [37].

Studies with longer followup and a larger number of patients are necessary to better define the role of these new and promising metabolic procedures in nonobese diabetic patients.

## 5. Conclusion

Inabnet et al. reported recently that, though patients with MetS presented with a similar preoperative weight profile as non-MetS patients (mean BMI 47 kg/m^2^), MetS patients experienced an increased incidence of serious complications, albeit infrequent, incidence of serious complications and mortality compared with non-MetS patients. Because the mean weight was similar between the two groups, factors other than weight invariably contribute to the greater incidence of adverse outcomes in MetS patients. For patients with MetS, the incidence of serious complications and mortality 90 days after bariatric surgery was 2.4% and 0.3%, respectively, significantly higher than non-MetS patients.

In this study, patients with MetS undergoing adjustable gastric banding had a lower incidence of mortality, serious complications, and readmissions compared with the other procedures; BPD/DS had the greatest incidence of adverse outcomes. The superior safety profile of adjustable gastric banding, however, was at the expense of decreased remission of hypertension, diabetes, dyslipidemia, and sleep apnea in patients with MetS [3]. These findings are consistent with the largest meta-analyses examining obesity and diabetes remission rates after bariatric surgery (Table 1) [38, 39]. 

In a recent review, Athyros et al. confirmed the efficacy of bariatric surgery on improvement of cardiovascular disease morbidity and mortality rate reduction. This effect appeared to result from an obesity-related comorbidities improvement. Among the different bariatric procedures, RYGBP was shown to be the most beneficial and have an acceptable safety profile [40]. Although this trend in improved efficacy across procedures tends to reflect the degree of weight loss and nutrient malabsorption induced by each procedure, recent evidence suggests that other factors including hormonal changes induced by bariatric surgery may play an important role in comorbidity resolution.

Type 2 diabetes is a medically incurable disease, which is often inadequately controlled. A growing body of evidence supports the new concept that type 2 diabetes is a surgically treatable “gut disease.” Diabetes surgery provides an entirely new opportunity to the study of pathophysiology of type 2 diabetes. The challenge for the next years will be the identification of criteria of patient's selection for “diabetic surgery” considering the “cost/effectiveness” on the basis of long-term results. The remaining pancreatic function should be tested as predictive criteria of long-term success. Studies to elucidate the role of caloric restriction versus weight loss, the role of the vagus nerve on gut peptide release, and the duodenal exclusion versus ileal exposure to nutrients are necessary to explain the mechanism of bariatric surgery on diabetes. Recent developments in experimental bariatric surgery such as ileal interposition and endoluminal procedure will require more research trials before becoming clinically applicable on a larger scale.

The results of experimental and clinical studies during the last decade have demonstrated that the manipulation of the gastrointestinal tract, inducing a rerouting of the food, induces relevant changes of the digestive hormonal pattern and finally affects the metabolic syndrome components. So this new experimental model of bariatric surgery gut manipulation represents an extraordinary and unique clinical model to study the physiopathology of the metabolic syndrome, mainly considering the gut involved in the etiopathogenesis of the T2DM in obese as well as in nonobese patients.

## Figures and Tables

**Table 1:** Bariatric surgery outcomes (weight loss and comorbidities resolution rate) (5–19, 39–42). Long term: followup > 3 years.

	LAGB		RYGBP		BPD-DS		LSG	
	1 year	Long term	1 year	Long term	1 year	Long term	1 year	Long term
% EWL	48	42.1	75	69	55	80	57.7%	66%
Hypertension	55%	56%	46%	81%	52.9%	40%	62.5%	85.7%
T2DM	58%	50%	72%	82%	74%	90%	76.9%	83%
Dyslipidemia	42%	40%	65%	40%	64.9%	44%	34%	80%
OSAS	45%	46%	75%	60%	44%	52%	56.2%	66%
